# MiRNA-21 Expression Decreases from Primary Tumors to Liver Metastases in Colorectal Carcinoma

**DOI:** 10.1371/journal.pone.0148580

**Published:** 2016-02-04

**Authors:** Fabian Feiersinger, Elke Nolte, Sven Wach, Tilman T. Rau, Nikolaos Vassos, Carol Geppert, Andreas Konrad, Susanne Merkel, Helge Taubert, Michael Stürzl, Roland S. Croner

**Affiliations:** 1 Division of Molecular & Experimental Surgery, Clinic of Surgery, Friedrich-Alexander- Universität Erlangen-Nürnberg, Schwabachanlage 12, 91054, Erlangen, Germany; 2 Clinic of Urology, Friedrich-Alexander-Universität Erlangen-Nürnberg, Krankenhausstr. 12, 91054, Erlangen, Germany; 3 Department of Pathology, Friedrich-Alexander-Universität Erlangen-Nürnberg, Krankenhausstr. 12, 91054, Erlangen, Germany; 4 Clinic of Surgery, Friedrich-Alexander-Universität Erlangen-Nürnberg, Krankenhausstr. 12, 91054, Erlangen, Germany; The University of Hong Kong, CHINA

## Abstract

**Objective:**

Metastasis is the major cause of death in colorectal cancer patients. Expression of certain miRNAs in the primary tumors has been shown to be associated with progression of colorectal cancer and the initiation of metastasis. In this study, we compared miRNA expression in primary colorectal cancer and corresponding liver metastases in order to get an idea of the oncogenic importance of the miRNAs in established metastases.

**Methods:**

We analyzed the expression of miRNA-21, miRNA-31 and miRNA-373 in corresponding formalin-fixed paraffin-embedded (FFPE) tissue samples of primary colorectal cancer, liver metastasis and healthy tissues of 29 patients by quantitative real-time PCR.

**Results:**

All three miRNAs were significantly up-regulated in the primary tumor tissues as compared to healthy colon mucosa of the respective patients (p < 0.01). MiRNA-21 and miRNA-31 were also higher expressed in liver metastases as compared to healthy liver tissues (p < 0.01). No significant difference of expression of miRNA-31 and miRNA-373 was observed between primary tumors and metastases. Of note, miRNA-21 expression was significantly reduced in liver metastases as compared to the primary colorectal tumors (p < 0.01).

**Conclusion:**

In the context of previous studies demonstrating increased miRNA-21 expression in metastatic primary tumors, our findings raise the question whether miRNA-21 might be involved in the initiation but not in the perpetuation and growth of metastases.

## Introduction

Colorectal cancer (CRC) is the third most frequent cancer in women and men, respectively [1]. The main cause of death from CRC is metastasis.[2] MicroRNAs (miRNAs) have been previously shown to be associated with disease progression and metastasis.[3–5] These short non-coding RNAs bind complementarily to the 3’-UTR of mRNA targets and thereby repress protein synthesis at the post-transcriptional level.[6] Several miRNAs are expressed differently in primary lesions of CRC as compared to uninvolved colorectal tissues [3]. Moreover, an association between altered miRNA expression and patient survival has been reported [7]. MiRNAs can foster CRC progression and metastasis by targeting pathogenetically important genes such as anti-oncogenes.

In this study, we focused on three miRNAs that may be crucial players in the formation of CRC metastasis: miRNA-21 has repeatedly been shown to be up-regulated in various cancers, targets the *tumor suppressor programmed cell death 4 (Pdcd4)* and may thus promote invasion, intravasation and metastasis.[8] MiRNA-31 exerts similar functions by suppressing *T-cell lymphoma invasion and metastasis-inducing protein 1 (TIAM1)*.[9] As yet, miRNA-373 has predominantly been in the focus of breast cancer research, but it increases the ability of cancer cells to migrate and invade by targeting CD44 [10, 11] and through this activity may also contribute to CRC progression.

To date, little is known about the role of miRNA expression in growth and perpetuation of already established metastases. In order to get a first estimate on this question, we compared the expression of miRNA-21, miRNA-31 and miRNA-373 in primary colorectal cancer (PrTu), healthy colorectal mucosa (HeMu), corresponding liver metastases (LiMe) and healthy liver specimens (HeLi) of the same patients, following the matched tissue approach suggested by de Krijger et al.[2] A relative decrease of miRNA-21 expression from PrTu to LiMe could indicate that the respective miRNA does not contribute to growth and/or perpetuation of the metastases.

Considering the restrictive patient and tissue sample inclusion criteria (cf. Materials and methods) and the matched tissue approach, 29 patients from a period of 14 years at the Department of Pathology at Friedrich-Alexander University Erlangen-Nürnberg could be selected for the study. So far, this is the largest cohort of the kind as for the analysis of miR-21 in colorectal cancer and corresponding liver metastasis.

## Materials and Methods

### Patient selection and tissue preparation

The study cohort comprised 29 patients. The study procedure was approved by the ethics committee at University Hospital Erlangen. According to the ethics committee, written consent was not necessary for this retrospective molecular analysis. All clinical data were analyzed anonymously. All work with human tissue was carried out in concordance with the World Medical Association's Declaration of Helsinki. Patient characteristics are summarized in Table 1. Follow-up data of patients were collected from the Erlangen Registry of Colorectal Cancer (ERCRC).

**Table 1:** Patient characteristics.

**1. General patient characteristics**	
**Median age in years (range)**	61,3 (43,5–79,3)
**Sex**	
Female	11 (38%)
Male	18 (62%)
**2. Characteristics of the primary tumor**	
**Location**	
Colon	15 (52%)
Rectum	14 (48%)
**UICC stage**	
I	3 (10%)
II	7 (24%)
III	7 (24%)
IV	12 (41%)
**pT classification (UICC 2009)**	
pT2	6 (21%)
pT3	20 (69%)
pT4a	2 (7%)
pT4b	1 (3%)
**pN classification**	
pN0	15 (52%)
pN+	14 (48%)
**Grading**	
G1	1
G2	20
G3	8
**Histological classification**	
Adenocarcinoma	27 (93%)
Mucinous adenocarcinoma	1 (3%)
Undifferentiated carcinoma	1 (3%)
**Lymphatic invasion (known for n = 28 patients)**	
yes	16
no	12
**Venous invasion (known for n = 28 patients)**	
yes	2
no	26
**Perineural invasion (known for n = 26 patients)**	
yes	7
no	19
**3. Characteristics of liver metastasis**	
**Time of diagnosis**	
Synchronous liver metastasis	12 (41%)
Metachronous liver metastasis	17 (59%)
**Grading of liver metastasis**	
G2	25 (86%)
G3	4 (14%)

Percentages not adding up to 100% are due to rounding.

In order to rule out any therapeutic influence on miRNA expression levels, none of the selected patients had undergone neoadjuvant radio- or chemotherapy before the operation on the primary tumor or on the corresponding LiMe. Four formalin-fixed and paraffin-embedded (FFPE) tissue specimens that had been created during routine pathology procedures were collected from each patient (PrTu, HeMu, LiMe and HeLi). All samples were drawn from the first surgical therapy on the respective disease. Surgery dates ranged from 1995 to 2008. The selection process was guided and independently validated by two experienced pathologists (TTR and CG). 2 μm sections of each sample were stained in hematoxylin-eosin (HE) and reviewed microscopically to identify tumor and healthy tissue. In addition, the share of tumor tissue on the section was determined. Only tumor and metastasis specimens with at least 30% tumor share were included. For RNA isolation, 10 μm sections were cut from each sample.

### RNA isolation and evaluation of RNA quality

Total RNA was isolated from 10 μm FFPE sections with a fully automated Siemens Tissue Preparation System and the VERSANT^®^ Tissue Preparation Reagents Kit (Siemens Healthcare Diagnostics, Tarrytown, USA), working with silica-coated iron oxide beads, as previously described.[12, 13] The first RNA quality evaluation step was DNA-specific quantitative PCR (qPCR) to test for possible DNA contamination. This was done with a primer/probe mix for the *progestagen-associated endometrial protein (PAEP)* gene (sequences in 5’-3’ orientation: probe: AAGCCCTCAGCCCTGCTCTCCATC, forward primer: CACAGAATGGACGCCATGAC, reverse primer: AAACCAGAGAGGCCACCCTAA). Then, the RNA content in the solutions was measured by quantitative reverse-transcription PCR (qRT-PCR) for the housekeeping gene *ribosomal protein L37a (RPL37a)* (sequences in 5’-3’ orientation: probe: TGGCTGGCGGTGCCTGGA, forward primer: TGTGGTTCCTGCATGAAGACA, reverse primer: GTGACAGCGGAAGTGGTATTGTAC). Primers and probes were custom-made by Eurogentec (Seraing, Belgium). For qRT-PCR reactions, we used the SuperScript^®^ III Platinum^®^ One-Step qRT-PCR kit with ROX (Invitrogen, Life Technologies, Darmstadt, Germany) according to the manufacturer’s instructions, except for a longer reverse transcription period of 30 min at 50°C. DNA-specific qPCRs were carried out with Platinum Taq DNA polymerase (Invitrogen). The PCR machine was a Stratagene/Agilent MX3005P QPCR system (Agilent, Waldbronn, Germany), a part of the Siemens VERSANT^®^ kPCR Molecular System.

### MiRNA expression analysis by qRT-PCR

TaqMan microRNA assays, cDNA synthesis and TaqMan reagents from Applied Biosystems (Foster City, USA) were used for miRNA quantification. QPCR was performed in triplicates in a StepOne plus real-time PCR system (Applied Biosystems). All reactions were carried out according to the manufacturer’s instructions, except for the following: the input amount of total RNA into cDNA synthesis was quantified by the results of RNA-specific qRT-PCR for the housekeeping gene *RPL37a*. Consequently, the linear range of miRNA-specific qRT-PCR had to be determined as a function of the C_t_ (cycle threshold) for *RPL37a*. This was done with dilution series with the miRNA-21 assay and the reference miRNA-16 assay (see below) for two samples with known C_t_ *(RPL37a)*, using the basic assumption that in quantitative PCR a 1:1 dilution equals a C_t_ increase by one.[14] With an input volume of 2 μl of total RNA into cDNA synthesis, qRT-PCR results were in a linear range from C_t_ (RPL37a) = 25 to C_t_ (RPL37a) = 21 with mean R^2^ for the two test samples of > 0.99 (miRNA-21) and > 0.98 (miRNA-16). As over 90% of the samples in the study collective showed a C_t_ (RPL37a) < 24, all those samples were diluted with nuclease-free water (Qiagen, Hilden, Germany) to a fictional C_t_ (RPL37a) = 24. 2 μl of this dilution were used for cDNA synthesis. For those samples with C_t_ (RPL37a) > 24, the input volume was adjusted accordingly to a maximum of 8 μl. Five samples exceeded the manufacturer’s constraint of a maximum of 5 μl of total RNA solution. To control whether this influenced qPCR results, mean C_t_ values for the two references (see below) were calculated over all samples and compared to the respective mean C_t_ values for these five samples. In miRNA-16, there was no difference with an overall mean C_t_ of 28.3 and a mean C_t_ of 28.2 for the five samples in question. In RNU6b, the five sample C_t_ mean was 33.8 in comparison to an overall mean C_t_ of 32.2.

### MiRNA-qPCR data normalization and statistical analysis

In qPCR, varying amounts of cells and total RNA in tissue samples require balancing by data normalization.[14] We used the NormFinder [15] algorithm to identify the reference miRNA with the most constant expression over all four tissue categories in this study (PrTu, HeMu, LiMe and HeLi). Three candidate references were tested with three samples from each category: miRNA-16 and miRNA-26a that have been shown to be stable between PrTu and HeMu [16] and RNU6b, a small nuclear RNA that has repeatedly been used to standardize miRNA expression values over different tissues [17–19]. With a stability value of 0.007, miRNA-16 was the most stably expressed miRNA in the test. As a combination of reference genes may be more appropriate than a single housekeeping candidate [20], miRNA-16 and RNU6b were both measured by qRT-PCR in the whole study collective and the geometric mean of the respective expression values was used for normalization.

Differences in miRNA expression levels between the four tissue categories were tested for with the Wilcoxon matched-pairs signed rank test. Mann-Whitney tests were used to analyze the association of miRNA-21 expression with histopathological data. Survival and the interval from PrTu diagnosis to LiMe in patients with metachronous metastases were analyzed with the Kaplan-Meier method; the Log-rank test was used to test for differences. P values < 0.05 were considered statistically significant. Statistical analysis was performed with GraphPad Prism 6 software (GraphPad software, La Jolla, USA).

## Results

In order to compare miRNA expression in colorectal cancer primary lesions, metastasis and the respective normal tissues, a careful standardization of analysis parameters was performed (Material and methods). The amount of RNA was normalized by adjusting all samples to a fixed C_t_ for the reference gene *RPL37a*. Then, the most stably expressed miRNA reference genes over the different tissues used in this study were identified. In order to further increase the reliability of normalization as compared to standardization against a single reference gene, the target miRNA expression was normalized against a combination of the two most stable reference genes, miRNA-16 and RNU6b.

Of the three miRNAs analyzed, miRNA-21 showed the highest expression with a mean C_t_ of 30.2 across all samples and tissue categories. MiRNA-31 und miRNA-373 were expressed at lower levels and were detected in only 21 and 19 patients, respectively.

### MiRNA expression in colorectal and liver specimens

**Fig 1:**
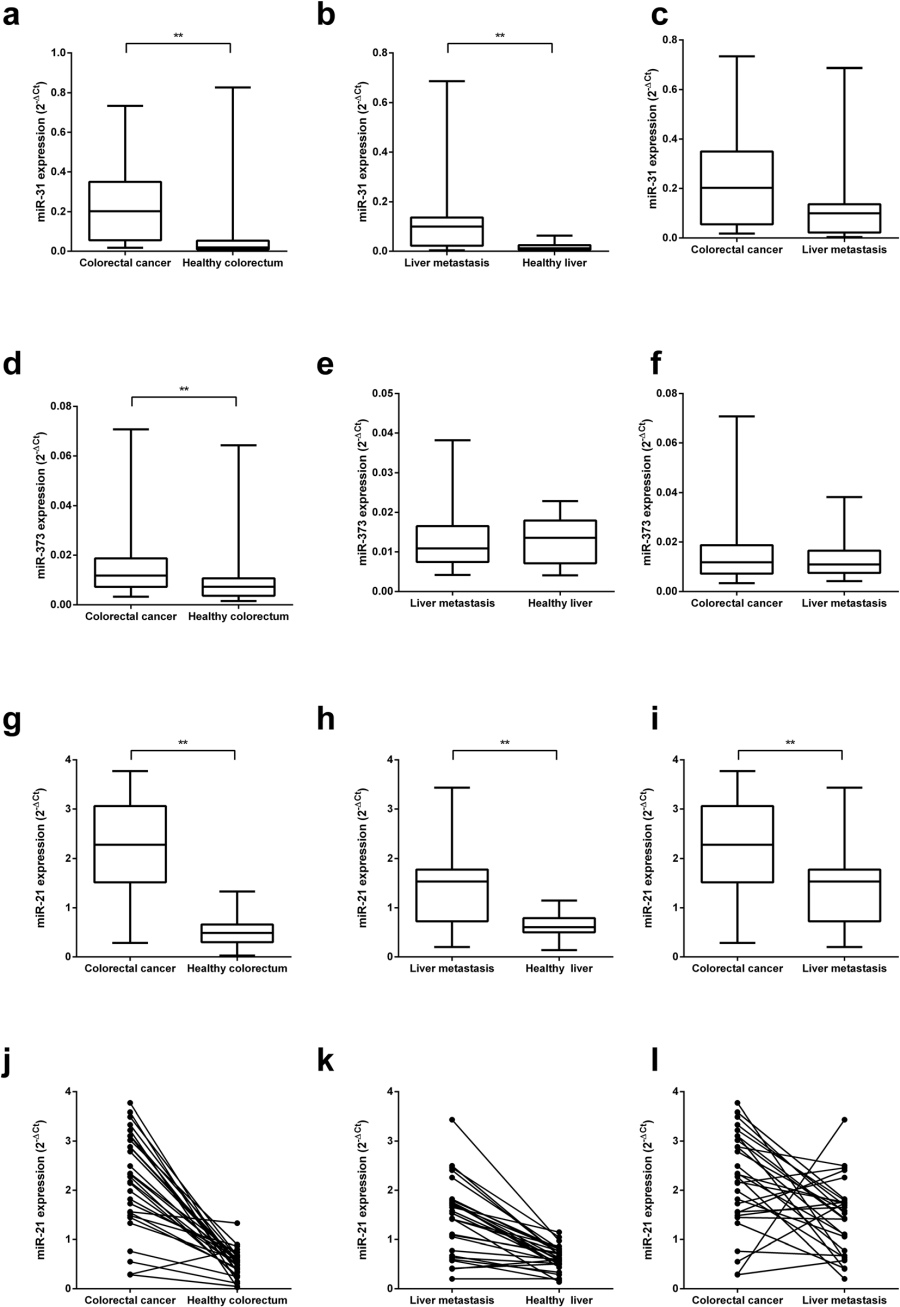
MiRNA expression levels in primary tumors, metastases and corresponding normal tissues of patients with colorectal carcinoma. **(a)–(i)** Aggregated box-plot comparisons of miRNA-31, miRNA-373 and miRNA-21 expression levels. All miRNAs are significantly higher expressed in colorectal carcinomas (PrTu) than in healthy colorectal mucosa (HeMu) **(a, d, g)**. Expression in liver metastases (LiMe) is significantly higher than in healthy liver tissue (HeLi) for miRNA-31 **(b)** and miRNA-21 **(h)**. A comparison of PrTu and LiMe shows significantly higher miRNA-21 in the primary tumor tissue (**i**; p < 0.01). MiRNA-31 is higher by trend (**c**; p = 0.09). **(j)–(l)** MiRNA-21 expression is compared on a combined intra- and inter-patient level over the same tissues. Expression levels in one patient are represented by two dots connected by a line. As for the comparison of PrTu and HeMu **(j)** and LiMe and HeLi **(k)**, respectively, in > 95% and about 90% of the patients, there is more miRNA-21 in the respective tumor than in the healthy tissues. **(l)** Comparison of miRNA-21 in PrTu and LiMe: 21 of 29 patients show higher miRNA-21 levels in the primary tumor. ** indicates p < 0.01.

MiRNA-31 was significantly up-regulated in PrTu (Fig 1a) and LiMe (Fig 1b) as compared with HeMu and HeLi, respectively (p < 0.01). It was also significantly higher in LiMe than in HeMu (p < 0.01). A slightly higher expression was detected in PrTu as compared to LiMe (Fig 1c), but this difference was not statistically significant (p = 0.09).

MiRNA-373 was significantly higher expressed in PrTu than in HeMu (Fig 1d; p < 0.01), too. However, no differences could be found between LiMe and HeLi as well as between PrTu and LiMe (Fig 1e and 1f).

MiRNA-21 was significantly up-regulated in PrTu as compared to HeMu (Fig 1g; p < 0.01), with a range of 1.2- to 120-fold. Expression was also significantly higher in LiMe than in HeLi (p < 0.01; Fig 1h). Up-regulation in metastases was from 1.2- to 10.3-fold. Of note, miRNA-21 expression was significantly lower in the liver metastases as compared to the primary tumors (p < 0.01; Fig 1i). At the single patient’s level, in 28 of 29 patients miRNA-21 expression was higher in the primary tumor tissue as compared to healthy colorectum (Fig 1j) and in 26 of 29 patients expression was higher in the liver metastasis as compared to healthy liver tissue (Fig 1k). Interestingly, in the majority of patients miRNA-21 expression was lower in the tumor tissues of the metastases as compared to the primary colorectal cancer tissue (21 of 29 patients, Fig 1l).

### Correlation of miRNA-21 expression with histopathological data and patient follow-up

**Fig 2:**
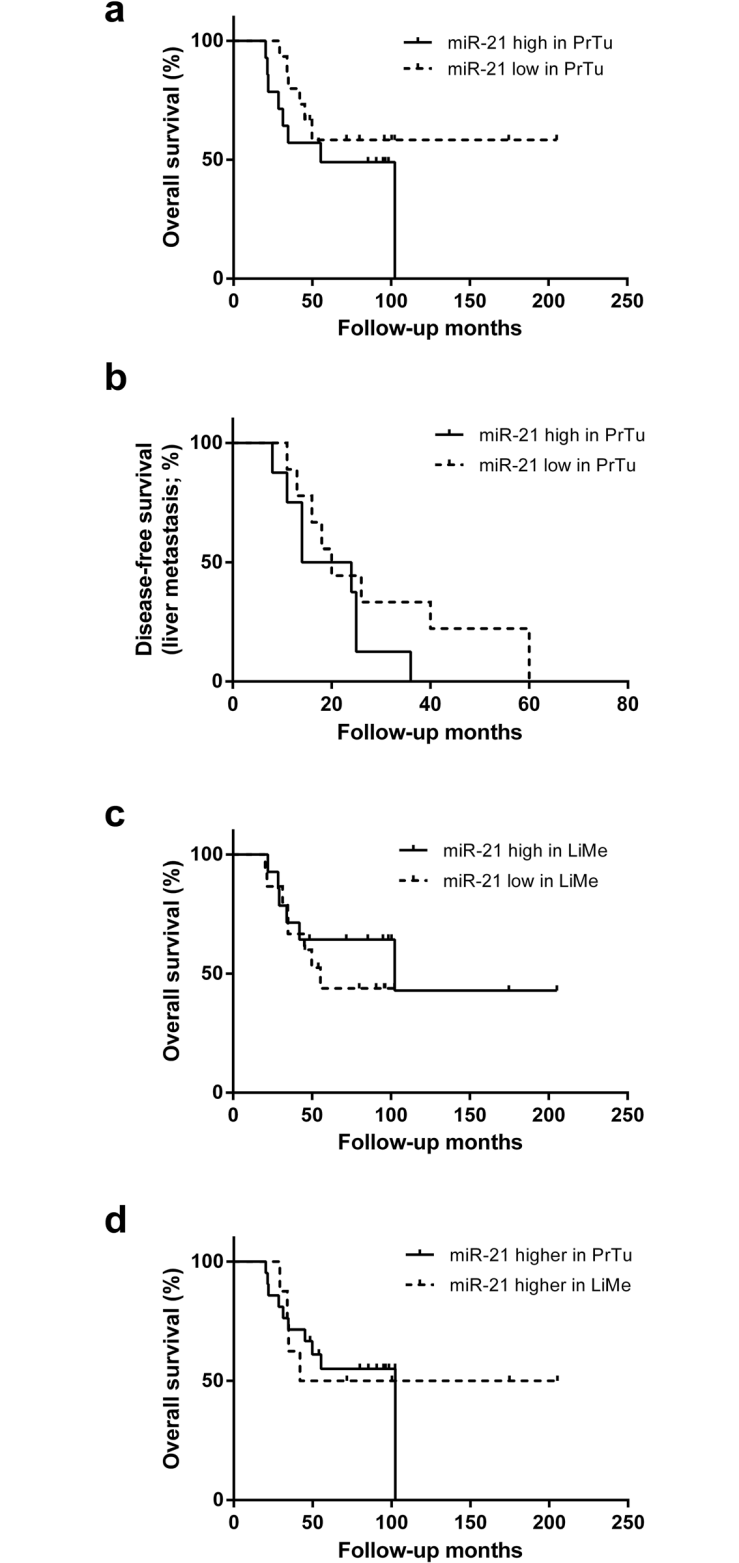
Correlation of miRNA-21 to patient follow-up. Disease-free survival with regard to liver metastasis (LiMe) and overall survival illustrated with the Kaplan-Meier method. **(a)** Comparison of overall survival between a miRNA-21 high and low expression group in colorectal carcinomas (PrTu). There is a trend towards shorter survival with higher miRNA-21 levels, although not significant (p = 0.27). **(b)** The patients with metachronous LiMe are sub-divided into a high and low miRNA-21 expression group in PrTu. By trend, high miRNA-21 led to earlier manifestation of LiMe (p = 0.20). **(c)** A comparison of survival between a high and a low miRNA-21 group in LiMe shows no significant difference (p = 0.44). **(d)** Survival does not differ significantly between those patients with higher miRNA-21 in the primary tumor and those with higher miRNA-21 in LiMe.

First, patients were classified according to above (high) and below (low) median miRNA-21 expression in the primary colorectal tumors. The level of miRNA-21 expression did not correlate significantly with tumor grading (p = 0.85), venous (p = 0.48), lymphatic (p > 0.99) or perineural (p > 0.99) invasion. Moreover, there was no correlation of miRNA-21 expression with lymph node metastasis (p = 0.93). A slight tendency towards shorter overall survival in patients with higher miRNA-21 levels in the primary tumor could be observed, although not significant (Fig 2a; p = 0.27). 17 patients in our study cohort suffered from metachronous LiMe. Comparing the interval from PrTu diagnosis to LiMe between the high and low miRNA-21 expression group showed a trend towards faster development of LiMe (p = 0.20; Fig 2b) with higher miRNA-21 levels in the PrTu.

No association of the miRNA-21 expression level in liver metastasis and overall survival was found (Fig 2c; p = 0.44). Moreover, the relative miRNA-21 expression level of the liver metastases as compared to the primary tumors was not associated with overall survival (Fig 2d, p = 0.86).

## Discussion

We performed a matched pair miRNA expression analysis in colorectal cancer and corresponding liver metastases from 29 patients using qRT-PCR. Great care was taken to establish reference genes for normalization. Using the NormFinder algorithm [15], the most stably expressed miRNA over all four tissue categories in this study was identified to be miRNA-16. In previous studies, this miRNA had already been suggested as a stable reference in colorectal cancer.[16] As combinations of reference genes may augment stability [20], we used the geometric mean of qRT-PCR expression values for miRNA-16 and RNU6b, which has repeatedly been used before to standardize miRNA expression values over different tissues [17–19], to normalize expression of the target miRNAs in this study. Moreover, RNA used for qRT-PCR was extracted from routinely processed FFPE material, showing that the procedure is suitable for a clinical application for the detection of miRNA.

MiRNA-31 and miRNA-373 had been previously related to colorectal cancer progression and metastasis in cell culture studies.[9–11, 21] At the tissue level, we found both miRNAs to be weakly expressed, which was in agreement with previous reports.[22], [23] Despite low expression, we found a significant up-regulation of both miRNA-31 and miRNA-373 in colorectal cancer specimens as compared to healthy colorectal mucosa. In addition, miRNA-31 was higher expressed in liver metastases than in healthy liver tissue.

The up-regulation of miRNA-21 in colorectal cancer tissue is an undisputed feature.[7, 24–26] We could confirm that the expression of miRNA-21 is increased in primary tumor tissues. In addition, we showed an increased expression in liver metastasis specimens as compared to the respective non-tumorous liver tissues of the same patients. In contrast, the influence of miRNA-21 on histopathology and the clinical course of colorectal cancer is controversial. A significant relationship between miRNA-21 expression and histopathological parameters, such as venous invasion, and overall survival had been reported [7, 26], but was not supported by others [22]. In agreement with the latter study, we did not find a significant correlation of miRNA-21 expression in the primary tumors with grading, lymph node metastasis, venous, lymphatic and perineural invasion. In concord with a previous study [24], a trend-like association of increased miRNA-21 expression in the primary tumors and reduced survival was observed, indicating that miRNA-21 expression may be associated with the initiation of metastases. Additionally, there was a trend towards faster development of liver metastasis in patients with higher miRNA-21 levels in the primary tumors. Taken together with the earlier finding that miRNA-21 levels are higher in metastatic primary tumors than in those that have not yet metastasized [24], miRNA-21 may be a candidate biomarker to estimate the risk level of CRC patients to develop metastasis. Biomarkers like this may be used in the future to adapt patient screening intensity according to individually estimated risk levels.[3]

While the oncogenic role of miRNA-21 in primary cancer formation is undisputed [7, 24–26], little is known about its features in already established metastasis. In our study, we followed the approach suggested by de Krijger et al., who emphasize the importance of comparing primary and matched metastatic tumor tissue from the same patients.[2] There are three existing publications that deal with miR-21 in primary CRC and colorectal liver metastases. One of these works with primary respectively metastatic tumor tissue from two different groups of patients [26], thus not following the above-mentioned important approach [2]. Here, no significant difference of miR-21 expression between PrTu and LiMe was found.[26] Drusco et al. and Vickers et al. analyze matched CRC and liver metastasis tissue samples from 17 and 19 patients, respectively. [24, 27] These works presented miRNA expression profiles that can differentiate between different types of colorectal metastases and metastatic and non-metastatic primary colorectal tumors. As for the expression of miR-21 in PrTu and LiMe, the former study found a trend towards higher miRNA levels in metastatic tissues, although not significant.[27] The latter did not present a significantly differential expression, either.

In our study, we aimed at a first assessment of a potential shift of importance of miR-21 over CRC progression: miR-21 overexpression has been associated with the formation of primary CRC [2, 7, 24–26] and corresponding metastasis [8, 9]. However, may there be a pattern change in metastasis after it has been successfully established? Altered expression of miR-21 in CRC metastases as compared to the primary tumors may be a first indicator of its oncogenic potential in established metastases. With an analysis of miRNA-21 expression in 29 cases of each primary CRC and matched healthy colorectal, liver metastasis and healthy liver tissue, we present the largest study cohort of the kind so far. MiRNA-21 expression could be shown to be significantly lower in liver metastases as compared to the primary tumors. This finding raises the question whether miRNA-21 may exert its pathogenic effects in the primary tumor tissues and the process of metastasis formation but might not contribute to the growth and perpetuation of CRC metastases.

Although we are able to present the largest patient cohort for an analysis of miR-21 expression in primary CRC and corresponding liver metastases, larger collectives need to be analyzed in order to confirm our finding. This can help to significantly reduce the influence of the immanent hetereogeneity of human samples. As a perspective, the expression of oncogenic miRNAs in primary tumors and corresponding metastasis should be evaluated in large cohorts of CRC patients with (liver) metastases. Since restrictive patient inclusion criteria, including the matched tissue approach, constrain the amount of patients that can be included into such a study, multi-center collaboration may be needed. Furthermore the functional role of miR-21 during tumor progression has to be elucidated in mechanistic studies to identify its value as potential therapeutic target in the future.

In addition, larger groups of colorectal cancer patients with metachronous liver metastases should be analyzed with regard to the relation of miRNA expression in the primary lesion and the time from the primary lesion to the diagnosis of metastasis. If the presented trend of higher miRNA-21 expression in the primary tumors leading to faster development of metastasis can be confirmed, this could further qualify miRNA-21 as an important biomarker.

## Supporting Information

S1 Table Raw data. (XLSX)

## Abbreviations

miRNA: microRNA
qRT-PCR: quantitative reverse-transcription polymerase chain reaction
CRC: colorectal cancer
3’ UTR: three prime untranslated region
mRNA: messenger RNA
PrTu: primary colorectal cancer
HeMu: healthy colorectal mucosa
LiMe: liver metastasis
HeLi: healthy liver tissue
FFPE: formalin-fixed paraffin-embedded
HE: hematoxylin-eosin
RPL37a: ribosomal protein L37a
cDNA: complementary DNA
C_t_: cycle threshold
